# Correlation between Exogenous Compounds and the Horizontal Transfer of Plasmid-Borne Antibiotic Resistance Genes

**DOI:** 10.3390/microorganisms8081211

**Published:** 2020-08-08

**Authors:** Yuan Liu, Ziwen Tong, Jingru Shi, Yuqian Jia, Kangni Yang, Zhiqiang Wang

**Affiliations:** 1 College of Veterinary Medicine, Yangzhou University, Yangzhou 225009, China; tongzw2019@163.com (Z.T.); Shijr2019@163.com (J.S.); jiayq2019@163.com (Y.J.); yangkn2019@163.com (K.Y.); 2 Institute of Comparative Medicine, Yangzhou University, Yangzhou 225009, China; 3 Jiangsu Co-innovation Center for Prevention and Control of Important Animal Infectious Diseases and Zoonoses, Joint International Research Laboratory of Agriculture and Agri-Product Safety, the Ministry of Education of China, Yangzhou University, Yangzhou 225009, China

**Keywords:** antibiotic resistance, compounds, conjugation, horizontal gene transfer, transformation

## Abstract

The global spread of antibiotic resistance has posed a serious threat to public healthcare and undermined decades of progress made in the fight against bacterial infections. It has been demonstrated that the lack of novel effective antibiotics and rapid spread of antibiotic resistance genes via horizontal transfer in the ecosystem are mainly responsible for this crisis. Notably, plasmid-mediated horizontal transfer of antibiotic resistance genes (ARGs) is recognized as the most dominant dissemination pathway of ARGs in humans, animals and environmental settings. Antibiotic selective pressure has always been regarded as one of the crucial contributors to promoting the dissemination of antibiotic resistance through horizontal gene transfer (HGT). However, the roles of exogenous compounds and particularly non-antibiotic drugs in the spread of ARGs are still underappreciated. In this review, we first summarize the major pathways of HGT in bacteria, including conjugation, transformation, transduction and vesiduction. Subsequently, an overview of these compounds capable of promoting the HGT is presented, which guides to the formulation of more reasonable dosing regimens and drug residue standards in clinical practice. By contrast, these compounds that display an inhibition effect on HGT are also highlighted, which provides a unique strategy to minimize the spread of ARGs. Lastly, we discuss the implementations and challenges in bringing these HGT inhibitors into clinical trials.

## 1. Introduction

The rapid emergence and dissemination of antibiotic resistance are increasing threats to public health [1,2]. It has been forecast that about 10 million lives would be lost due to infections caused by multidrug resistant bacteria in 2050 if the current situation continues [3]. Importantly, plasmid-mediated intra- and inter-species horizontal gene transfer (HGT) is commonly acknowledged as a major driver for the prevalence and spread of antibiotic resistance genes (ARGs) in the environment, human beings and animals [4,5]. For instance, since *bla*_NDM-1_ gene-mediated carbapenems resistance was first identified in 2009 [6], this gene has been widely reported in clinically relevant pathogens from human and animal sources [7]. Additionally, the mobilized colistin resistance gene *mcr-1-*positive Enterobacteriaceae [8] from different origins has been identified in over 50 countries across six continents. Meanwhile, various *mcr-1* variants, such as *mcr*-*2/3/4/5/6/7/8/9/10*, were also identified in bacteria from various sources [9]. Previous research has shown that sub-minimum inhibition concentrations (MIC) of specific antibiotics can profoundly facilitate the conjugation process, thus improving the relatively low conjugation efficiency in the experimental conditions [10]. However, other compounds, such as environmental contaminants, also play a crucial role in the dissemination of ARGs, whereas their actions are largely neglected. Therefore, extensive attention should be paid to these exogenous compounds that contribute to HGT. A better understanding of the effect and molecular mechanisms of these non-antibiotic drugs on HGT would be conducive to developing more effective strategies to control the spread of ARGs. Meanwhile, the effect of these potential inhibitors of HGT is also worthy of remark as it provides a distinct pipeline in combating antibiotic resistance.

In this review, we provide an overview of HGT pathways and their key determinants. In addition, we systematically summarize these compounds that could promote or inhibit HGT in bacteria and outline their underlying molecular mechanisms. Finally, implications and perspectives for control of the dissemination of ARGs in ecological niches were highlighted.

## 2. Horizontal Transfer of Antibiotic Resistance Genes

HGT generally refers to genetic communication between individuals of different species that can cross reproductive isolation barriers. Essentially, HGT represents the process of sharing genes or genetic material among individuals of different species, which adds new genetic variation to the recipient organisms and avoids the destructive effect caused by the gradual accumulation of point mutations. In addition, dominant traits in the process of life evolution can spread rapidly among individuals of different species, aiding them to quickly adapt to the new environment or obtain new resources [11]. Before widely found in eukaryotes, HGT was first identified in prokaryotes, which are smaller, easier to spread and highly adaptable to the environment, particularly in bacteria [12]. Because most point mutations are not beneficial or harmful to individuals, and prokaryotes lack heritable variation caused by sexual reproduction, HGT is an important means to obtain new genetic material for them. In addition to obligate endosymbiont bacteria, most bacteria quickly adapt to the new environment by obtaining genes from other species in the environment, which is often accompanied by the loss of other genes [13]. Altogether, HGT has been considered as the main driving force of prokaryotic evolution regardless of some debates.

**Figure 1:**
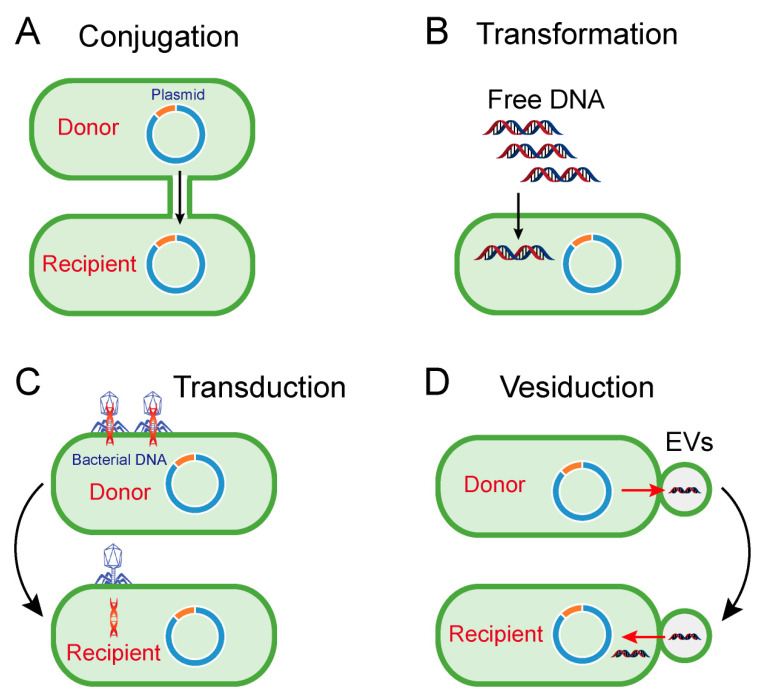
Pathways of horizontal gene transfer. (**A**) Conjugation is the process of DNA transfer from donor cell to recipient cell via cell-to cell contact. (**B**) Transformation represents the uptake and integration of naked fragments of extracellular DNA by recipient cells. (**C**,**D**) Transduction and vesiduction refer to the transfer of bacterial DNA (red DNA strand) by bacteriophages (C) or extracellular vesicles (EVs, D).

Accordingly, conjugation, transformation, transduction and vesiduction were recognized as four major pathways for HGT in prokaryotes (particularly bacteria) (Figure 1) [14,15]. Among these four pathways, conjugation is commonly considered as the most dominant route [16]. Differently from transformation and transduction, conjugation requires cell-to-cell contact via cell surface pilus or adhesions, offering better protection from the disturbed environment and a more efficient means to transfer bacterial genes from a donor cell to a recipient cell (Figure 1A) [17]. Differently from the sex pilus, which helps the donor cell attach to the recipient cell produced by the donor cells in Gram-negative bacteria, the contact in Gram-positive bacteria relies on surface adhesins [18]. After the connection, the two cells will directly contact and form a coupling bridge by which DNA can be transferred from the donor to the recipient. Generally, this process includes two steps: (1) DNA is mobilized by *mob* genes-encoded relaxase proteins; (2) single-strand DNA is transported via a type IV secretion system (T4SS) [19].

Although conjugation could also occur in some Gram-positive bacteria, the modes of action, particularly in cell recognition, are different from those in Gram-negative bacteria [18]. Similarly, the conjugation process in both Gram-negative and Gram-positive bacteria is highly dependent on the conjugative plasmids-encoding conjugation machinery. Meanwhile, conjugation machinery enables the transfer of non-conjugative plasmids, such as IncQ plasmids, with a broad range of hosts [20]. Notably, these mobile genetic elements, which included antibiotic resistance genes located in plasmids or chromosomes, are transferred from environment-derived bacteria to various human health-relevant pathogens [21]. Moreover, transfer of ARGs between unrelated bacteria with large taxonomic distances was also identified [22,23]. These examples suggest the remarkable role of conjugation in the dissemination of ARGs within different reservoirs.

Transformation refers to the process that extracellular free fragments of DNA are taken up and integrated by certain bacteria (Figure 1B) [24]. It requires several conditions, including free DNA fragments and competent recipient bacteria, and the translocated DNA must be integrated into the recipient genome or encircled as plasmid DNA [15]. In order to effectively absorb DNA, bacterial cells must be in a competent state, as defined by the ability of bacteria to bind to free fragments of DNA, and are formed only in a limited number of bacteria, such as *Haemophilus* and *Streptococcus* [25]. However, many other bacteria, such as *E. coli*, can be artificially rendered to be competent under specific stressful conditions, such as antibiotic stress or calcium chloride (CaCl_2_) stimulation [26]. Consistently, it has been indicated that antibiotic treatment could promote the transformation of extracellular DNA [27]. It is also noteworthy that the transformation of ARGs has been widely found in various species. For example, the fluoroquinolone resistance gene (*gyrA*) and penicillin resistance gene (*penA*) could be transformed into *S. pneumoniae* and commensal *Neisseria* species, respectively [28,29].

Unlike conjugation and transformation-mediated HGT, transduction is performed by bacteria-infecting viruses termed bacteriophages. In this process of genetic recombination, genes from host cells are first incorporated into the genome of the bacteriophage, and then carried to the recipient cell after the next bacteriophage infections (Figure 1C) [30]. Transduction is a reliable way to transfer DNA between bacteria because it can protect foreign DNA enclosed in bacteriophages from physical degradation and DNase in the environment. Additionally, cell-to-cell contact, which is indispensable in conjugation, is not necessary for transduction. However, bacteriophages-mediated transduction is not applicable for horizontal transfer of extensive genes owing to the inefficient package of bacteriophages and its high specificity to infected bacteria. Therefore, interspecific transfer caused by transduction is not common in the environmental setting. Nevertheless, several studies have suggested that bacteriophages-mediated transductions also partially account for the prevalence of ARGs. For instance, the presence of ARGs in bacteriophages has been detected in cystic fibrosis patients [31], wastewater samples [32] and animal and human fecal samples [33], suggesting that bacteriophages are potential reservoirs of ARGs.

In addition to the three canonical mechanisms above, extracellular vesicles (EVs)-mediated DNA transfer, termed vesiduction (Figure 1D), has been proposed as a fourth mode of HGT [34]. The secretion of EVs from bacterial membranes was first observed in the 1960s [35], and subsequently proven as a widespread phenomenon in both Gram-positive and Gram-negative bacteria [36,37,38,39]. Moreover, eukaryotes and archaea such as hyperthermophilic and halophilic archaea can also produce EVs [40], suggesting that this biological phenomenon is not specific to prokaryotes. It has been evidenced that EVs are associated with different physiological roles [41,42], which are highly dependent on their composition. For example, peptidoglycan hydrolases or toxin-containing EVs are involved in pathogenicity or competition with other microorganisms; quorum sensing molecules may correlate in cell-to-cell communications [38]. Additionally, EVs can function as DNA carriers capable of protecting DNA from degradation by restriction enzymes, DNase or other physical and chemical conditions, thus playing a crucial role in HGT. In 2000, Yaron et al. first indicated that EVs from *E. coli* O157:H7 are responsible for the transfer of virulence genes [43]. Thereafter, EVs were found to transfer endogenous and/or exogenous plasmids from cell to cell [40,44]. All examples shed light on the fact that vesiduction-conferred HGT is becoming increasingly common in diverse microorganisms. However, the precise mechanisms of vesiduction remain obscure, especially when it comes to secretion and its regulatory factors of EVs, as well as its recognition and fusion with recipient cells.

## 3. Exogenous Compounds Promote Horizontal Genes Transfer

A series of investigations have revealed the high incidence of AGRs in bacteria from humans [45], animals and environmental samples [46,47]. Considering the relatively low HGT frequency between bacteria in drug-free conditions, exogenous compounds, such as environmental pollutants, may be conductive to accelerating this progress (Table 1). Hence, a better understanding of these incentives would contribute to developing more integrated strategies to defer the spread of ARGs.

It has been widely acknowledged that antibiotic residue would promote the process of HGT, particularly for ARGs located in plasmids whose horizontal transfer mainly stems from the increased expression of conjugation machinery induced by antibiotics [48]. For example, cefotaxime treatment significantly increased the conjugation frequency of *bla*_CTX−M−1_-bearing IncI1 resistance plasmid in a reference strain *Escherichia coli* MG1655 [49]. Proteome analysis revealed that the expression of conjugation associated proteins such as Tra drastically up-regulated after exposure to cefotaxime. Gentamicin was also found to promote the conjugation between *E. coli* and *Pseudomonas aeruginosa* by blocking quorum sensing [50]. Various studies demonstrated that non-antibiotic drugs play a catalytic role in HGT. For instance, disinfectants including free chlorine, chloramine and hydrogen peroxide promoted the transfer of the resistance genes *sulI* and *aadA2* from the *intI1*-positive bacterium *Aerococcus* sp. to the *intI1*-negative bacterium *Pseudoalteromonas* sp [51]. Preservatives (sodium nitrite, sodium benzoate and triclocarbon) [52], under daily use concentrations, resulted in dose-dependent increases in conjugative transfer frequency of ARGs. Evidence showed that these preservatives could activate radical-induced RpoS regulon and SOS response, enhance cell membrane permeability and upregulate conjugative transfer-related genes, subsequently promoting the horizontal transfer of ARGs. Recently, the antiepileptic drug carbamazepine at environmentally relevant concentrations was found to promote the conjugative transfer of ARGs mediated by RP4 plasmid within and across bacterial genera [53]. To figure out the underlying correlation, mechanistic studies were performed, illustrating that carbamazepine indeed promoted the production of reactive oxygen species (ROS), enhanced cell membrane permeability and increased the expression of conjugation-related genes. Meanwhile, the non-antimicrobial agent triclosan was confirmed to facilitate the horizontal transfer of ARGs through both conjugation [54] and transformation [55].

Nanomaterials possess unique physicochemical and biological properties, and have been experimentally proven to be versatile in many fields, including cancer treatment, antibacterial application, drug delivery and drug residue detection [56,57,58]. Nevertheless, the effect of nanomaterials on the transfer of ARGs between bacteria remains obscure. It has been reported that nanoalumina significantly enhanced the horizontal conjugative transfer of multidrug-resistance genes-bearing RP4, RK2 and pCF10 plasmids from *E. coli* to *Salmonella* spp. with an approximate 200-fold change [59]. Mechanistic studies also showed that nanoalumina could trigger oxidative stress, damage bacterial cell membranes, enhance the expression of DNA transfer and replication genes, and depress the expression of global regulatory genes.

**Figure 2:**
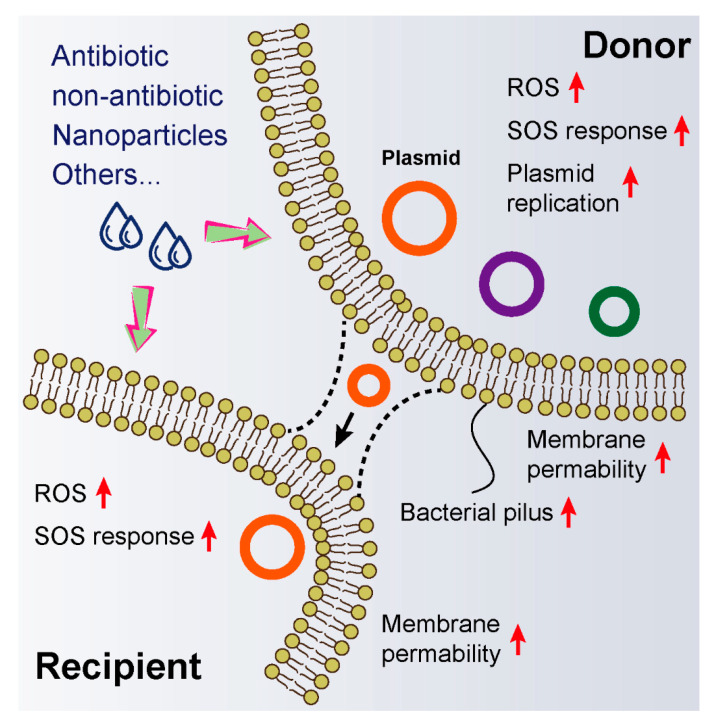
Schematic representation of molecular mechanisms that exogenous compounds promote—conjugative transfer of resistance plasmids. Exogenous compounds including antibiotics, non-antibiotic drugs, nanoparticles and others could promote horizontal gene transfer within or across bacterial genera through triggering the production of ROS, inducing SOS response, enhancing membrane permeability, increasing bacterial pilus generation and promoting plasmid replication.

Besides, other compounds such as ionic liquids (ILs), which have been widely applied in the chemical industry as a replacement of industrial volatile organic solvents, were found to facilitate the proliferation of ARGs in environmental bacteria [60,61,62]. Two typical polycyclic aromatic hydrocarbons (naphthalene and phenanthrene) significantly enhanced the abundance of class I integrase gene (*intI1*), aminoglycosides resistance gene (*aadA2*) and sulfanilamide resistance gene (*sulI*) in the microbial community via promoting conjugative transfer. Interestingly, a recent study showed that CO_2_ promoted the conjugative transfer of multi-resistance genes by increasing proton motive force (PMF), thus providing new insight into public health issues under global warming [63]. Altogether, these compounds remarkably promote the horizontal transfer of antibiotic resistance genes through common mechanisms, including over-production of ROS, SOS response, enhanced membrane permeability, increased expression of plasmid replication genes and generation of bacterial pilus (Figure 2)**.** However, the specific molecular mechanisms of these actions and their relationships remain obscure. Gene knockout experiments and corresponding phenotype analysis may be conducive to elucidating the underlying pathways. Nevertheless, these findings strengthen our understanding of the dissemination of antibiotic resistance that is enhanced by non-antibiotic pharmaceuticals, thus alerting us to rethink the reasons for the spread of resistance genes in the environment.

## 4. Exogenous Compounds Inhibit Plasmid Transfer

Plasmid curing and anti-plasmid approaches provide a unique strategy to minimize the prevalence of ARGs in a variety of natural environments. A recent study demonstrated that inhibiting plasmid conjugation was an effective means to remove resistance plasmid from a bacterial population over time and reduce AMR plasmid prevalence [64]. Apart from these methods, novel strategies also sprung up in recent decades, including conjugation or transformation inhibitors, plasmid incompatibility systems, CRISPR/Cas-based approaches and phages [65,66,67,68].

**Figure 3:**
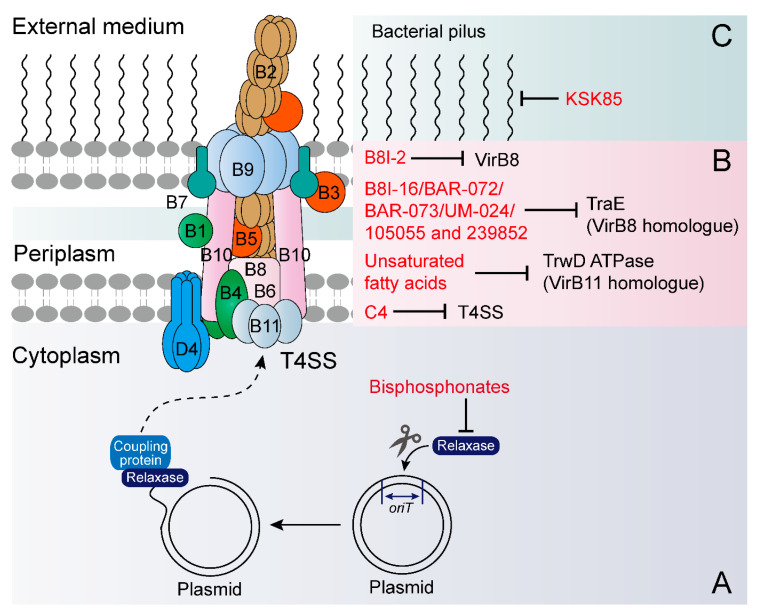
Promising targets of HGT inhibitors on conjugative machinery. Conjugative relaxase protein encoded by the *mob* gene cleaves plasmid strands at the origin of transfer (oriT) and covalently binds to the DNA at the 5′ end. With the assistance of the coupling protein, this nucleoprotein complex is recruited at the secretion channel (T4SS). (**A**) Conjugative relaxase protein initiates conjugation upon nicking plasmid DNA at the origin of transfer, and thereby has been considered as a potential target for HGT inhibitors. (**B,C**) The transportation of DNA is mediated by a type IV secretion system (T4SS), a protein complex (also known as a mating pore formation (MPF)) that consist of substrate transport (**B**) and pilus biogenesis (**C**). Generally, substrate transport comprises 11 proteins, named VirB1 to VirB11. Thus, these proteins and conjugative pilus could be a potential molecular target for HGT inhibitors. 105055, (4-(1H-pyrrol-1-yl)pyridine-2-carboxylic acid); 239852, (2-(2-furyl)isonicotinic acid).

In this review, we put emphasis on drug-dependent means, including conjugation and transformation inhibitors (Table 2). Bottom-up strategies by which screening compounds target bacterial secretion machinery offer a feasible approach to identify novel conjugation inhibitors. One potential target is the *mod*-encoded conjugative relaxase protein, which initiates conjugation upon nicking plasmid DNA at the beginning of a transfer [69]. Through cell-based assays, bisphosphonates (clodronate and etidronate) were found to prevent conjugative DNA transfer by inhibiting the conjugative DNA relaxase (Figure 3A) [70].

Another typical target is the VirB protein, which is an indispensable assembly protein of bacterial T4SS (Figure 3B) [71]. A high throughput assay for restoring the interaction between two split domains of the *Brucella* VirB8 protein enabled the discovery of novel compounds that inhibited protein–protein interactions [72]. One of the most efficient molecules, named B8I-2 (a salicylidene acylhydrazide derivative), is also known to inhibit T3SS [73]. Subsequent X-ray crystallography coupled with in silico docking of several of these compounds revealed their accurate binding sites in the VirB8 protein [74]. Using a targeted approach, a study revealed that some small molecules can bind to TraE, a homolog of VirB8 that is an essential component of all T4SSs, and some of them effectively inhibited pKM101 plasmid transfer [75]. However, no inhibition effect of these molecules on the conjugation of control plasmid RP4 was observed, suggesting their influence is plasmid-specific. In a follow-up study, Casu et al. screened a focused fragment library for compounds that target TraE, which promoted the design and obtainment of two potential compounds (molecules 105055 and 239852) that could bind to TraE with high affinity and reduce transmission of pKM101 [76].

Fatty acids have been proven to be effective conjugation inhibitors on a variety of resistance plasmids. For example, four unsaturated fatty acids (linoleic, oleic, 2-hexadecynoic and 2-ocatadecynoic acid) could inhibit the activity of TrwD ATPase (VirB11 homologue) by binding to the linker region and *N*-terminal domain of TrwD, eventually affecting bacterial conjugation [77]. Additionally, synthetic fatty acids, particularly 2-hexadecynoic acid, decreased conjugation frequencies of IncW, IncH and IncF plasmids by 100 times through acting on the donor bacteria, including *E. coli*, *S. enterica*, *P. putida* and *Acinetobacter baumannii* [78]. However, the toxicity of these synthetic fatty acids in people or animals acts as the biggest barrier to their clinical use and renders them ambiguous in reliability. To avoid this defect, less-toxic natural compounds, such as tanzawaic acids isolated from *Penicillium* species, were instead identified as effective conjugation inhibitors of IncW and IncFII plasmids [79].

Considering the requirement of conjugative pili for cell contact in Gram-negative bacteria [80], the use of specific compounds to inhibit pilus formation exhibits its possibility and prospect in the fight against this problem. As was reported, two peptidomimetic molecules (KSK85 and C10) disrupted T4SS-dependent processes in multiple bacterial pathogens [81]. Specifically, KSK85 impeded biogenesis of the pilus appendage associated with the *cag* T4SS (Figure 3C), while C10 disrupted cagT4SS activity without perturbing pilus assembly. Consequently, these compounds prevented T4SSs-mediated interbacterial DNA transfer in conjugative *E. coli*.

**Figure 4:**
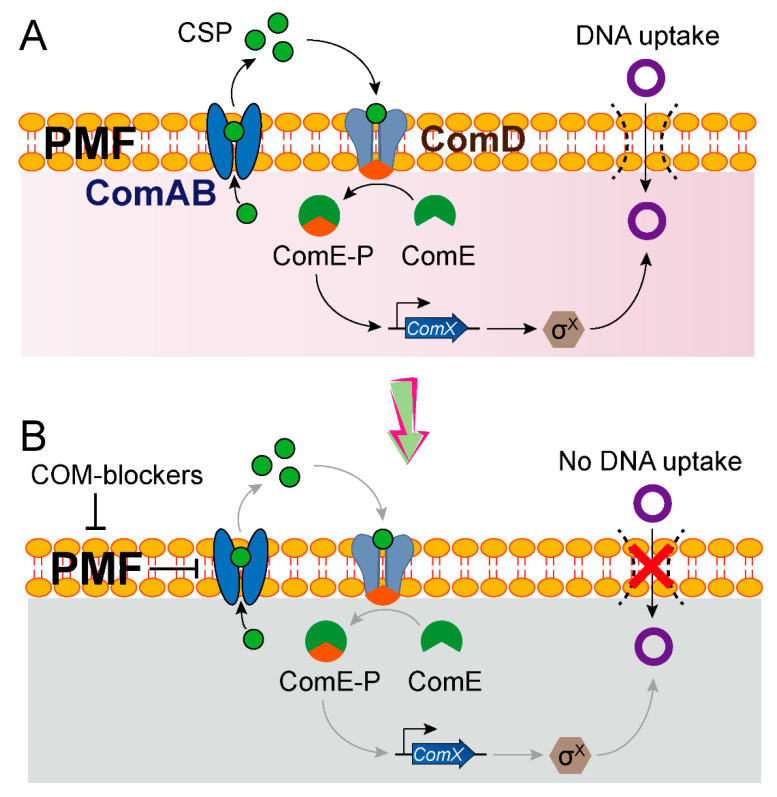
COM-blockers limit the HGT in *S. pneumoniae* by inhibiting the proton motive force (PMF) [84]. (**A**) Overview of the competence-dependent regulatory mechanism of HGT in *S. pneumoniae*. The membrane transporter ComAB first exports competence-stimulating peptide (CSP) with the aid of proton motive force (PMF). Then, CSP binds to histidine-kinase ComD, which accounts for the transfer of phosphate group to the downstream response regulator ComE. Phosphorylated ComE, in turn, promotes the expression of SigX encoded by *comX*, which is responsible for the uptake of exogenous DNA. (**B**) COM-blockers, including the biocide triclosan, the antimalarial proguanil hydrochloride and the antipsychotic pimozide, displayed the ability to inhibit PMF and block the secretion of CSP, thereby preventing the uptake of DNA and HGT.

In addition to targeting the direct DNA conjugation machines by corresponding inhibitors, the transformation inhibitors are of equal significance in preventing the spread of ARGs. As described above, since competent state-mediated transformation machinery is necessary for the uptake and integration of exogenous DNA, inhibitors of competence would contribute a lot to preventing the spread of resistance genes. In *S. pneumoniae*, two key operons, including *comCDE* and *comA*, are involved in competence activation (Figure 4A) [82]. The membrane transporter ComAB is responsible for cleaving and exporting the comC-encoded CSP (competence-stimulating peptide), which results in the phosphorylation of response regulator ComE that in turn activates competence related genes. Fully intact PMF involved in F0F1 ATPase activity is indispensable for downstream ComD-ComE signaling and natural competence induction [83]. Based on these points, a recent study performed a high-throughput screening and discovered three potent inhibitors of *S. pneumoniae* competence (Figure 4B) [84], called COM-blockers. These COM-blockers, including the biocide triclosan, the antimalarial proguanil hydrochloride and the antipsychotic pimozide, limited bacterial competence by blocking the proton motive force (PMF), thus preventing the export of a quorum-sensing peptide that regulates the transformation machinery. Eventually, COM-blockers were confirmed to inhibit HGT both in vitro and in vivo. Interestingly, it seems contradictory that triclosan was found to simultaneously promote and inhibit transformation based on different mechanisms in different studies. Thus, further studies are requisite to figure out the underlying reasons.

Notably, some other promising HGT inhibitors have been reported, but the mechanism by which these compounds prevent the transfer of resistance genes-bearing plasmid remains unclear. For instance, anti-HIV agents, such as abacavir and azidothymidine, were demonstrated to inhibit the transfer of plasmids carrying carbapenemase and extended spectrum β-lactamase genes [85]. In addition, our study revealed that azidothymidine prevented the conjugation of *tet*(X4)-bearing plasmid in *E. coli* [86]. Hence, it would be worthwhile to systematically explore the inhibition effect of azidothymidine on other resistance genes or plasmid types. Importantly, the underlying mechanisms are expectable and might give aid to the development of new HGT inhibitors. In addition, numerous studies have put forward that nanomaterials may promote horizontal gene transfer, while rare reports about the inhibition effect of nanomaterials on HGT have been mentioned. Recently, Fe_2_O_3_@MoS_2_ was found to inhibit the conjugative transfer of RP4-7 plasmid by increasing the expression of the global regulatory gene (*trbA*) and reducing the expression of conjugative transfer genes (*traF*, *trbB* and *trfA*) [87]. Meanwhile, flavophospholipol [88] and isothiocyanates [89] displayed an inhibitory effect on the horizontal transfer of various plasmids types through blocking bacterial conjugation, whereas the underlying molecular mechanisms remain obscure.

## 5. Conclusions and Perspectives

Antibiotic resistance is spreading at a fast rate within pathogenic bacteria and infections caused by antibiotic-resistant bacteria have become an urgent issue for global health. Simultaneously, the lack of novel effective antibiotics and rapid spread of antibiotic resistance genes via horizontal transfer in the ecosystem exacerbate this crisis beyond doubt.

To counter this dilemma and avoid getting trapped in tougher circumstances in which no effective antimicrobial agents could be used, a comprehensive understanding of the effect of exogenous compounds on HGT, including promotion or inhibition functions, is therefore urgently required. In this review, we first provided an overview of four pathways that account for the horizontal transfer of antibiotic resistance genes, including conjugation, transformation, transduction and vesiduction. Then, we summarized these compounds that could promote the horizontal transfer of plasmid-borne multi-antibiotic resistance genes within and across bacterial genera. Altogether, the underlying mechanisms comprise over-production of ROS, activation of the SOS response, enhanced cell membrane permeability and generation of bacterial pilus. These important findings undoubtedly ring alarm bells for us to re-think and re-evaluate the potential detriment of non-antibiotic compounds in environmental settings on the prevalence of ARGs. Additionally, further studies remain warranted to verify whether these non-antibiotic drugs are able to promote conjugation in more complex ecosystems, such as in sludge, soils or human guts.

Compared with compounds that promote HGT, the inhibitors of HGT seem to be more or less neglected in spite of their potential to be excavated. As is known, some potent inhibitors, such as unsaturated fatty acids, have exhibited an effective antagonism effect on the conjugation of a variety of plasmids in in vitro experiments. However, the in vivo safety and efficacy of these inhibitors have not been completely evaluated. Meanwhile, a series of additional problems should be seriously considered before bringing these candidates into clinical trials [90]. For example, who would like to use the HGT inhibitors, patients or environmental settings? Is the patient willing to take this compound that appears to have no beneficial effect on infections? Will the use of these drugs in the environment cause potential environmental pollution and other unknown problems? Despite these challenges, HGT inhibitors might serve as potential candidates for resistance plasmid curing strategies, thereby offering a promising approach to control the dissemination of ARGs based on the concept of “One Health”.

## Figures and Tables

**Table 1:** Exogenous compounds that promote horizontal transfer of antibiotic resistance genes (ARGs).

Compounds	Year	Species	Plasmid/ARGs	Mechanisms
Silver ions/nanoparticles	2020	*E. coli* → *P. putida*	RP4	ROS generation, membrane damage and the SOS response.
Triclosan	2020	Plasmid → *E. coli*	pUC19	Triggers ROS over-production, damages cell membrane barrier, mediates the pilus capture of plasmid and the translocation of plasmid via cell membrane channels.
Preservatives	2020	*E. coli* → *E. coli*	pCM194-Cm	Stimulates radical-induced RpoS regulon and SOS response, increases cell membrane permeability, and regulates conjugative transfer-related genes.
Copper nanoparticles/ions	2019	*E. coli* → *P. putida*	RP4	Over-production of ROS.
Carbamazepine	2019	*E. coli* → *E. coli*; *E. coli* → *P. putida*;	--	Increases ROS and the SOS response and enhances cell membrane permeability and pilus generation.
CO_2_	2019	*E. coli* → *E. coli*; *E. coli* → *S. typhimurium*;	RP4	Reduces intercellular repulsion and increases PMF.
Triclosan	2018	*E. coli* → *E. coli*; *E. coli* → *P. putida*;	RP4	Promotes ROS generation and damages bacterial membrane, and causes increased expression of the SOS response regulatory genes.
Disinfectants (free chlorine, chloramine, and hydrogen peroxide)	2017	*E. coli* → *E. coli*; *E. coli* → *S. typhimurium*;	--	Intracellular ROS formation, SOS response, increases cell membrane permeability, and alters expressions of conjugation relevant genes.
Polycyclic aromatic hydrocarbons (PAHs)	2017	*intI1*-positive bacterium *Aerococcus* sp. → *intI1*-negative bacterium *Pseudoalteromonas* sp.	*intI1*, *sulI* and *aadA2* genes	--
Gentamicin	2017	*E. coli* → *P. aeruginosa*	RP4	Inhibits quorum sensing.
Ionic liquid ((BMIm)(PF6))	2015	*E. coil* K12 → *Salmonella* spp.; *E. coil* K12 → *microbacterium* spp.	RP4	Enhances cell membrane permeability.
Ionic liquid ((BMIm)(PF6))	2014	*intI*-positive bacteria to other bacterial strains	*sulI* and *intI* genes	Increases cell membrane permeability.
Nanoalumina	2012	*E. coli* → *Salmonella*; *E. coli* → *E. coli*; G^+^ → G^−^; *Enterococci* → *Enterococci*	RP4	Damages bacterial membranes, enhances the expression of conjugative genes and represses global regulatory factor genes.

ARGs, antibiotic resistance genes; G^+^, Gram-positive bacteria; G^−^, Gram-negative bacteria; ROS, reactive oxygen species; PMF, proton motive force; --, not applicable.

**Table 2:** Exogenous compounds that inhibit horizontal transfer of antibiotic resistance genes (ARGs).

Compounds	Year	Species	Plasmid	ARGs	Mechanisms
COM-blockers (triclosan, hydrochloride and pimozide)	2020	*S. pneumoniae* → *E. coli*	--	--	Inhibits PMF, thereby preventing the export of a quorum-sensing CSP
Azidothymidine	2020	*E. coli* → *E. coli*	Tet(X)-producing plasmids	*tet*(X3/X4)	--
Anti-HIV drugs (abacavir and azidothymidine)	2020	*E. coli* → *E. coli K. pneumoniae* → *K. pneumoniae*	ESBL-producing plasmid (pCT) and carbapenemase-producing plasmid (pKpQIL)	Extended spectrum β-lactamase and carbapenemase genes	--
Fe_2_O_3_@MoS_2_	2019	*E. coli* → *E. coli*; *E. coli* → *E. faecalis*; *E. faecalis* → *E. faecalis*	RP4-7	--	Promotes the expression of global regulatory gene (*trbA*) and inhibits the expression of conjugative transfer genes
Isothiocyanates	2019	*E. coli* → *E. coli*	pKM101 (IncN), TP114 (IncI_2_), pUB307 (IncP) and R7K (IncW)	--	--
Flavophospholipol	2019	*E. coli* → *E. coli*; *E. faecalis* → *E. faecalis*	Self-transmissible plasmids carrying the βlactamase genes or *vanA* genes	Extended-spectrum β-lactamase and *vanA* genes	--
Molecules (105055 and 239852)	2017	*E. coli* → *E. coli*	pKM101	--	Targets the TraE protein
Unsaturated fatty acids	2016	*E. coli* → *E. coli*	IncW, IncH, IncF IncI, IncL/M and IncX	--	Targets type IV traffic ATPase TrwD
Tanzawaic acids	2016	*E. coli* → *E. coli*	IncW and IncFII	--	--
B8I-16, BAR-072, BAR-073 and UM-024	2016	*E. coli* → *E. coli*	pKM101	--	Targets the TraE protein
Peptidomimetic	2016	*E. coli* → *E. coli*	IncF plasmid	--	Disrupts type IV secretion system
Synthetic fatty acids	2015	*Escherichia, Salmonella, Pseudomonas* and *Acinetobacter* spp	IncF, IncW, IncH, IncI, IncL/M, and IncX plasmids	--	--
Bisphosphonates	2007	*E. coli* → *E. coli*	F plasmid	--	Inhibits the conjugative DNA relaxase

ARGs, antibiotic resistance genes; COM, competence; CSP, competence-stimulating peptide; ESBL, extended-spectrum β-lactamases; 105055, (4-(1H-pyrrol-1-yl)pyridine-2-carboxylic acid); 239852, (2-(2-furyl)isonicotinic acid); --, not applicable.
